# A Review of 25 Spontaneous and Dynamic Facial Expression Databases of Basic Emotions

**DOI:** 10.1007/s42761-024-00289-3

**Published:** 2025-01-15

**Authors:** Hyunwoo Kim, Yifan Bian, Eva G. Krumhuber

**Affiliations:** Department of Experimental Psychology, University College London, 26 Bedford Way, London, WC1H 0AP UK

**Keywords:** Emotion, Facial expression, Dynamic, Spontaneous, Database

## Abstract

Most prior research on basic emotions has relied upon posed, static displays that do not accurately reflect the facial behavior seen in everyday life. To address this gap, the present paper aims to highlight existing facial expression databases (FEDBs) that feature spontaneous and dynamic displays of the six basic emotions. To assist readers in their decisions about stimulus selection, we comprehensively review 25 FEDBs in terms of three key dimensions: (a) *conceptual features* which reflect thematic approaches in database construction and validation, i.e., emotional content and elicitation procedures, encoder demographics, measurement and elicitation techniques; (b) *technical features* which concern technological aspects in stimulus development, i.e., stimulus numbers and duration, frame rate, and resolution; and (c) *practical features* which entail information about database access and potential ethical restrictions. Finally, we outline some of the remaining challenges in stimulus generation and make recommendations for future research.

Facial expressions play an integral role in socio-cognitive and affective processes, such as in emotion recognition (Krumhuber et al., 2023), empathy formation (Israelashvili et al., 2019), and social perception (Jack & Schyns, 2015). Basic emotions as proposed by Basic Emotion Theory (Ekman & Friesen, 1975)—happiness, sadness, anger, fear, disgust, and surprise—have long been a cornerstone in the study of emotions and facial expressions (Ekman, 1992, 1999; Keltner et al., 2019). Numerous works have shown that specific facial patterns of these emotions can be reliably recognised and discriminated by human judges (for reviews see Brosch et al., 2010; Calvo & Nummenmaa, 2016). The wide use of basic emotions is evident from the substantive number of articles published in major journals in fields such as psychology, neuroscience, and computer science (Table 1). To date, research on basic (compared to non-basic) emotions provides the largest corpus of scientific evidence available (Durán & Fernández-Dols, 2021). Moreover, the concept remains highly influential in the field of affective computing, with the majority of automatic facial expression analysis (AFEA) tools attempting to recognise primary affective states of basic emotions (for reviews see Calvo et al., 2010; Zeng et al., 2009).

**Table 1:** Percentage and number of articles using facial expression stimuli of the six basic emotions for major journals in fields such as psychology, neuroscience, and computer science^1^

Journal	% of articles ^a^	No. of articles	% of articles using different types of facial expression stimuli ^b^			
			Posed	Spontaneous	Static	Dynamic
*Affective Science*	64%	9	78%	22%	67%	33%
*Behaviour Research Methods*	58%	7	57%	43%	29%	71%
*Cognition and Emotion*	77%	36	75%	8%	75%	19%
*Emotion*	78%	43	84%	12%	56%	37%
*Frontiers in Psychology*	44%	71	76%	13%	63%	25%
*Journal of Experimental Psychology*	100%	4	75%	25%	100%	0%
*Journal of Experimental Social Psychology*	60%	3	100%	0%	100%	0%
*Journal of Personality and Social Psychology*	30%	3	100%	0%	67%	0%
*Plos ONE*	35%	49	61%	24%	57%	41%
*Psychological Science*	44%	4	75%	25%	75%	25%
*Scientific Reports*	61%	51	67%	27%	63%	31%
*Social Cognitive and Affective Neuroscience*	70%	7	100%	0%	86%	14%
*Transactions on Affective Computing*	87%	13	46%	54%	23%	69%

^1^The search syntax consisted of keywords related to ‘spontaneous and dynamic’ and ‘basic emotion’ in combination with ‘facial expression database’. Keywords were interlaced using the Boolean operators ‘AND’ and ‘OR’, thereby enhancing the scope of the search. To concentrate efforts on highly relevant articles, we constrained our search to titles, abstracts, and keywords represented by ‘TITLE-ABS-KEY’, whilst the truncation symbol ‘*’ was deployed to accommodate all derivatives of a keyword (e.g., happ*: happy, happiness). The ellipses ‘…’ indicate the positions where additional synonyms for each keyword were placed in the search query. Subject area and journal titles were specified with ‘SUBJAREA’ and ‘EXACTRCTITLE’, respectively. The following search syntax was used:

(TITLE-ABS-KEY(spontaneous*) …) AND (TITLE-ABS-KEY(dynamic*) …) AND (TITLE-ABS-KEY(anger*) OR TITLE-ABS-KEY(disgust) OR TITLE-ABS-KEY(fear*) OR TITLE-ABS-KEY(happ*) OR TITLE-ABS-KEY(sad*) OR TITLE-ABS-KEY(surprise)) AND (TITLE-ABS-KEY(face*) OR TITLE-ABS-KEY(facial*) …) AND (TITLE-ABS-KEY(express*) OR TITLE-ABS-KEY(display*) …) AND (TITLE-ABS-KEY(database*) OR TITLE-ABS-KEY(corpus*) …) AND PUBYEAR > 2020 AND PUBYEAR < 2024 AND (LIMIT-TO(SUBJAREA, “subject area”)) AND (LIMIT-TO(EXACTSRTITLE, “Journal name”))

*Note*. ^a^*N* articles using facial expression stimuli of the six basic emotions divided by *N* total number of articles returned by Scopus (including irrelevant articles) for each journal for 2020–2024. ^b^Summed percentages of posed and spontaneous stimuli, and static and dynamic stimuli may add up to < 100% because some articles did not specify the type and format of display

Unfortunately, most prior research on basic emotions has relied upon posed and static images—typically captured at expression apex (Table 1; see also Dawel et al., 2021). Whilst offering high experimental control, this approach has sparked concerns regarding the use of exaggerated and stereotypical displays (Kim et al., 2023; Matsumoto & Hwang, 2017; Nelson & Russell, 2013). With the growing interest in spontaneous facial expressions, a number of stimulus sets have emerged in recent years, allowing crucial insights into more lifelike behavior paired with higher ecological validity. This paper aims to provide a systematic and topical review of spontaneous and dynamic facial expression databases (FEDBs) of basic emotions. By detailing their conceptual and technical features, we aim to highlight commonalities as well as differences between the datasets, thereby assisting the research community in making well-informed decisions about stimulus selection.

Spontaneous facial expressions encompass a wide range of involuntary facial displays elicited through experimental manipulations (e.g., picture/video viewing, olfactory stimulation, imagination, memory retrieval) that resemble emotionally evoking situations in the real world (Weber et al., 2018). Because they occur without any instructions what emotion/behavior to produce, spontaneous expressions depict (unlike posed displays) more accurate representations of affective responses seen in everyday life. At the same time, they allow for sufficient experimental control over contextual variations (e.g., background, noise, lighting, and recording angle), which distinguishes them from naturalistic ‘in-the-wild’ expressions recorded in unstructured/unconstrained environments.

In terms of facial features, spontaneous expressions are less prototypical and more ambiguous due to the expressive variability between senders (Barrett et al., 2019). A recent meta-analysis revealed that over 75% of spontaneous expressions of the six basic emotions are non-prototypical (Durán & Fernández-Dols, 2021). The lack of common prototypical patterns makes them challenging to recognise, with classification rates typically ranging from 15 to 65% for human observers (Kayyal & Russell, 2013; Wagner, 1990) and 50 to 70% for AFEA (Dupré et al., 2020; Krumhuber et al., 2021; Liu et al., 2020). Importantly, dynamic motion benefits the recognition of spontaneous expressions by offering distinct temporal information for emotion discrimination (Krumhuber et al., 2023).

Motivated by the need for ecologically valid facial stimuli, a number of FEDBs have been created over the past years. To inform the research community, several papers offer a comparative review of selected corpora, but those typically feature a mixture of expressions including static and posed displays (Corneanu et al., 2016; Diconne et al., 2022; Guerdelli et al., 2022; Haamer et al., 2018; Jia et al., 2021; Krumhuber et al., 2017; Siddiqui, 2022; Weber et al., 2018). Also, they are limited in the range of spontaneous datasets surveyed, with many of the FEDBs discussed in this paper not being part of previous reviews. To address these limitations, the present paper aims to provide a comprehensive and systematic review targeting spontaneous, dynamic FEDBs of basic emotions and highlighting key dimensions and properties of the available datasets. Nearly half of these databases feature in addition to the basic emotions (including contempt) also non-basic emotional and mental states.

To provide readers with essential information about each dataset, we classified databases in terms of three key dimensions and attributes: (a) *conceptual features* (Table 2), which reflect thematic approaches in database construction and validation; (b) *technical features* (Table 3), which concern technological aspects in stimulus development; and (c) *practical features* (Table 4), which entail information about database access.

**Table 2:** Conceptual features of 25 spontaneous and dynamic facial expression databases

Database	Expression		Encoder demographics				Measurement and validation				
	Emotions	Elicitation	Ethnicity/nationality	Age	Gender	*N*	Annotation	FACS coding	Classifiers	Measures	Self-report
BAUM-1 (Zhalehpour et al., 2017)	6 basic emotions, boredom, contempt, confusion, thinking, concentration, bothered, neutral	Movies, video ads, TV shows, IAPS images	Turkish	19–65	18 M, 13 F	31	Emotions		AFEA	Emotions	
BINED (Sneddon et al., 2012)	6 basic emotions, frustration	Films, wire tracking task, touching objects	Irish, Peruvian	> 18	137 M, 119 F	256	Emotions, valence				Emotions
BioVid Emo (Zhang, Walter et al., 2016)	Anger, disgust, fear, amusement, sadness	Films	German	18–65	44 M, 50 F	86					Emotions, valence, arousal
BP4D-Spon (Zhang et al., 2014)	Happiness/amusement, sadness, surprise/startle, anger/upset, fear/nervous, disgust, embarrassment, pain	Video, joke, insult, startle probe, unpleasant smell, cold pressor task, singing, dart game	Asian, Black, white, Hispanic	18–29	18 M, 23 F	41	AUs	Y	AFEA, human observers	Emotions, AUs	Emotions
BP4D + (Zhang, Girard et al., 2016)	Happiness/amusement, sadness, anger, disgust, startle/surprise, joyful surprise, skeptical, fear/nervous, embarrassment, pain	Video, joke, insult, startle probe, unpleasant smell, cold pressor task, singing, dart game, true/false question, self-avatar	Asian, Black, White, Hispanic, others	18–66	58 M, 82 F	140	AUs	Y	AFEA	Emotions, AUs	Emotions
BP4D + + (Li et al., 2023)	Happiness/amusement, sadness, anger, disgust, startle/surprise, joyful surprise, skeptical, fear/nervous, embarrassment, pain	Video, joke, insult, startle probe, unpleasant smell, cold pressor task, singing, dart game, true/false question, self-avatar	Asian, Black, White, Hispanic, others	18–70	101 M, 132 F	233	AUs	Y	AFEA	AUs	Emotions
ChildEFES (Negrão et al., 2021)	6 basic emotions, contempt, neutral	Cartoon excerpts	Caucasian, African, Asian	4–6	51 M, 73 F	124	Emotions		Human observers	Emotions	
DECAF (Abadi et al., 2015)	Amusement/happiness/funniness, excitement, anger, disgust, fear, sadness, shock	Movies, music videos		*M* = 27.3	16 M, 14 F	30			AFEA	Emotions	Valence, arousal
DISFA (Mavadati et al., 2013)	Joy, disgust, fear, sadness, surprise	YouTube videos	White, Black, Hispanic, Asian	18–50	15 M, 21 F	27	AUs	Y	AFEA	AUs	
DynEmo (Tcherkassof et al., 2013)	Cheerfulness, curiosity, pride, astonishment, disgust, moved, boredom, fright, annoyance, shame, neutral	Videos, video ads, pictures, defective software, positive feedback, false belief	Caucasian	25–65 (*M* = 48)	176 M, 182 F	358			Human observers	Emotions	Emotions, action readiness, valence, arousal
EB + (Ertugrul et al., 2020)	Happiness/amusement, sadness, anger, disgust, startle/surprise, joyful surprise, skeptical, fear/nervous, embarrassment, pain	Video, joke, insult, startle probe, unpleasant smell, cold pressor task, singing, dart game, true/false question, self-avatar	Asian, Black, White, Hispanic, others	18–66	82 M, 118 F	200	AUs	Y	AFEA	Emotions, AUs	Emotions
Emognition (Saganowski et al., 2022)	Amusement, anger, awe, disgust, enthusiasm, fear, liking, sadness, surprise, neutral	Films	Polish	19–29 (*M* = 22.4)	22 M, 21 F	43		Y	AFEA	Emotions, AUs	Emotions, valence, arousal, motivation
FEEDTUM (Wallhoff et al., 2006)	6 basic emotions, neutral	Films	White/Caucasian			19			AFEA, human observers	Emotions	
I.Vi.T.E. (Esposito et al., 2012)	Happiness, fear, sadness, disgust, neutral	YouTube videos	Italian	21–28		49	Emotions				
iSAFE (Singh & Benedict, 2020)	6 basic emotions, uncertainty, no emotion	Videos	Indian	17–22	25 M, 19 F	44	Emotions	Y	AFEA	Emotions, AUs	Emotions
ISED (Happy et al., 2015)	Happiness, surprise, sadness, disgust	Movies	Indian	18–22	29 M, 21 F	50	Emotions		AFEA	Emotions	Emotions
KTFE (Nguyen et al., 2014)	6 basic emotions, neutral	Videos, game	Thai, Japanese, Vietnamese	11–32	16 M, 10 F	26	Emotions		AFEA	Emotions	Emotions
KTFEv2 (Nguyen et al., 2023)	6 basic emotions, neutral	Videos, game	Thai, Japanese, Vietnamese	11–32	19 M, 11 F	30	Emotions		AFEA	Emotions	Emotions
LIRIS-CSE (Khan et al., 2019)	Disgust, fear, happiness, sadness, surprise	Cartoon videos, movies	Various ethnicities	6–12 (*M* = 7.3)	5 M, 7 F	12	Emotions		AFEA, human observers	Emotions	
NVIE (Wang et al., 2010)	6 basic emotions	Films	Chinese	17–31	157 M, 58 F	315	Emotions, valence, arousal		AFEA	Emotions, valence, arousal, genuineness	Emotions, valence, arousal
PEDFE (Miolla et al., 2022)	6 basic emotions	Films, video ads, YouTube videos, unpleasant smell, computer games	White/ Caucasian	20–30		56	Emotions, AUs	Y	Human observers	Emotions, genuineness	Emotions, genuineness
PPB-Emo (Li et al., 2022)	6 basic emotions, neutral	Videos	Chinese	19–58 (*M* = 28.1)	31 M, 9 F	40					Emotions, valence, arousal, dominance
SASE-FE (Kulkarni et al., 2021)	Anger, disgust, happiness, sadness, surprise, contempt	YouTube videos	White/Caucasian, Asian, African	19–36	32 M, 22 F	54			AFEA	Emotions	
SPOS (Pfister et al., 2011)	6 basic emotions	Films	White/Caucasian, Asian		4 M, 3 F	7	Emotions		AFEA	Valence, genuineness	Emotions
UT-Dallas (O’Toole et al., 2005)	6 basic emotions, boredom, disbelief, laughter, puzzlement, neutral	Movies, TV programmes	Asian, Black, White, Hispanic, Other	18–25	76 M, 208 F	284	Emotions				

*Note. N* refers to encoder numbers after exclusion. ‘Measures’ describes the variables being measured by human observers or AFEA (so-called ‘Classifiers’) to assess the recognition/judgment of stimuli. Whilst stimuli can be assessed or undergo FACS coding, they may not be annotated with data labels in those terms. On the other hand, data annotators may assign labels to stimuli which have not been measured by Classifiers or through self-report

**Table 3:** Technical features of 25 spontaneous and dynamic facial expression databases

Database	*N* (videos/frames)	Duration	Frame rate	Resolution
BAUM-1	1184	.43 to 9.34 s (*M* = 1.82)	30 fps	576 × 720
BINED	1400	5 to 180 s		720 × 576, 1920 × 1080
BioVid Emo		32 to 245 s (*M* = 68)	25 Hz	1388 × 1038
BP4D-Spontaneous	328	~ 1 min	25 fps	1040 × 1392
BP4D +	1.4M frames		25 fps	1040 × 1392
BP4D + +	> 94K frames		25 fps	1040 × 1392
ChildEFES	702	10 to 60 s		720
DECAF		51.1 to 128.2 s	20 fps	
DISFA	130K frames	4 min	20 fps	1024 × 768
DynEmo	358	1 to 15 min	25 fps	768 × 576
EB +	1261	44 s	25 fps	1040 × 1392
Emognition	430	49 s to 2 min	60 fps	1920 × 1080
FEEDTUM	399	30 to 300 s	25 fps	640 × 480
I.Vi.T.E	120K frames		1 fps	640 × 480
iSAFE	395		60 fps	1920 × 1080
ISED	428	1 to 10 s	50 fps	1920 × 1080
KTFE			5 fps	
KTFEv2			5 fps	
LIRIS-CSE	208	*M* = 5 s	25 fps	720 × 480, 800 × 600, 1920 × 1080
NVIE		3 to 4 min	30 fps	704 × 480
PEDFE	707	*M* = 3.0 s	30 fps	
PPB-Emo	240	30 s	30 fps	1920 × 1080
SASE-FE	~ 324	3 to 4 s	100 fps	1280 × 960
SPOS	147	*M* = 13 s	25 fps	640 × 480
UT-Dallas		5 s	30 fps	720 × 480

**Table 4:** Practical features of 25 spontaneous and dynamic facial expression databases

Database	Website	Email address	Access	Payment
BAUM-1	http://archive.ics.uci.edu/ml/datasets/BAUM-1	s.zhalehpour@gmail.com; cigdem.erogluerdem@gmail.com	Email	
BINED		g.mckeown@qub.ac.uk	Email	
BioVid Emo	https://www.nit.ovgu.de/BioVid.html	sascha.gruss@uni-ulm.de	EULA form	
BP4D-Spontaneous	https://www.cs.binghamton.edu/~lijun/Research/3DFE/3DFE_Analysis.html	lijun@cs.binghamton.edu	Email	$400
BP4D +	https://www.cs.binghamton.edu/~lijun/Research/3DFE/3DFE_Analysis.html	lijun@cs.binghamton.edu	Email	$1,250
BP4D + +		lijun@cs.binghamton.edu	Email	
ChildEFES		juliana@negrao.co	Email	
DECAF	https://decaf-dataset.github.io/	decaf.mhug@gmail.com	EULA form	
DISFA	http://mohammadmahoor.com/disfa/	mmahoor@du.edu	EULA form	
DynEmo	https://dynemo.univ-grenoble-alpes.fr/?page=inscription	anna.tcherkassof@upmf-grenoble.fr	Email	
EB +	https://www.cs.binghamton.edu/~lijun/Research/3DFE/3DFE_Analysis.html	lijun@cs.binghamton.edu	Email	$500
Emognition	https://github.com/Emognition/Emognition-wearable-dataset-2020	emotions@pwr.edu.pl	EULA form	
FEEDTUM	https://www.jade-hs.de/team/frank-wallhoff/databases/	frank.wallhoff@jade-hs.de	EULA form	
I.Vi.T.E		Iiass.annaesp@tin.it	Email	
iSAFE	https://github.com/shivendra2015iiit/Indian-Semi-Acted-Facial-Expression-Database-iSAFE-	shivendra15@alumni.iiitkottayam.ac.in	Email	
ISED	https://sites.google.com/site/iseddatabase/	iseddatabase@gmail.com	EULA form	
KTFE		hungnv@hcmue.edu.vn	Email	
KTFEv2		hungnv@hcmue.edu.vn	Email	
LIRIS-CSE	https://childrenfacialexpression.projet.liris.cnrs.fr	rizwan.khan@bhu.edu.pk	EULA form	
NVIE	https://ustc-ac.github.io/datasets/nvie/	sfwang@ustc.edu.cn	EULA form	
PEDFE	https://osf.io/cynsx/	alessio.miolla@phd.unipd.it	OSF	
PPB-Emo		guogang@cqu.edu.cn	Email	
SASE-FE		shb@icv.tuit.ut.ee	EULA form	
SPOS	https://www.oulu.fi/en/university/faculties-and-units/faculty-information-technology-and-electrical-engineering/center-machine-vision-and-signal-analysis	Xiaobai.Li@oulu.fi	EULA form	
UT Dallas	https://labs.utdallas.edu/facelab/database/	otoole@utdallas.edu	EULA form	

## Systematic Review Protocol

The literature search involved three focal search engines/databanks: *PsycInfo*, *Web of Science*, and *SCOPUS*, spanning the period from 1990 to September 2023. To broaden the search spectrum, supplementary platforms such as Google Scholar and AI-integrated search tools (including Elicit.org) were also used. We further undertook a manual examination of previous review papers and reference lists pertaining to FEDBs for potential inclusion in this work.

The search syntax was developed from keywords synonymous with ‘spontaneous’ and ‘dynamic facial expression databases’, which were interlaced using ‘AND’ and ‘OR’ Boolean operators. The resulting search syntax was as follows: (spontaneous OR natural OR genuine OR authentic OR real OR involuntary OR induced) AND (dynamic OR move OR moving OR motion OR action OR video OR image sequence) AND (anger OR disgust OR fear OR happiness OR sadness OR surprise) AND (face OR facial OR emotional OR affect OR nonverbal OR physical) AND (expression OR behaviour OR display OR visage OR presentation OR manifest OR feature OR communication) AND (database OR data set OR corpus OR corpora OR collection). Field codes used to specify search areas could vary across search engines (e.g., [tiab] for PsycINFO, [TITLE-ABS-KEY] for SCOPUS).

All retrieved articles were subsequently imported into Zotero reference management software (Fairfax, Virginia, USA) to systematically handle the search results and streamline the removal of duplicates across various sources. The literature selection protocol adhered strictly to the Preferred Reporting Items for Systematic Reviews and Meta-Analysis (PRISMA; Page et al., 2021) guidelines, a structured approach offering a robust framework for systematic reviews. Using a stepwise approach for filtering appropriate literature, we followed four main steps: (1) sourcing literature that described spontaneous FEDBs of basic emotions from diverse sources, (2) a preliminary screening of titles and abstracts to gauge their relevance, (3) an exhaustive full-text review to confirm eligibility, and (4) finalising the selection of FEDBs to be included in this review.

**Fig. 1:**
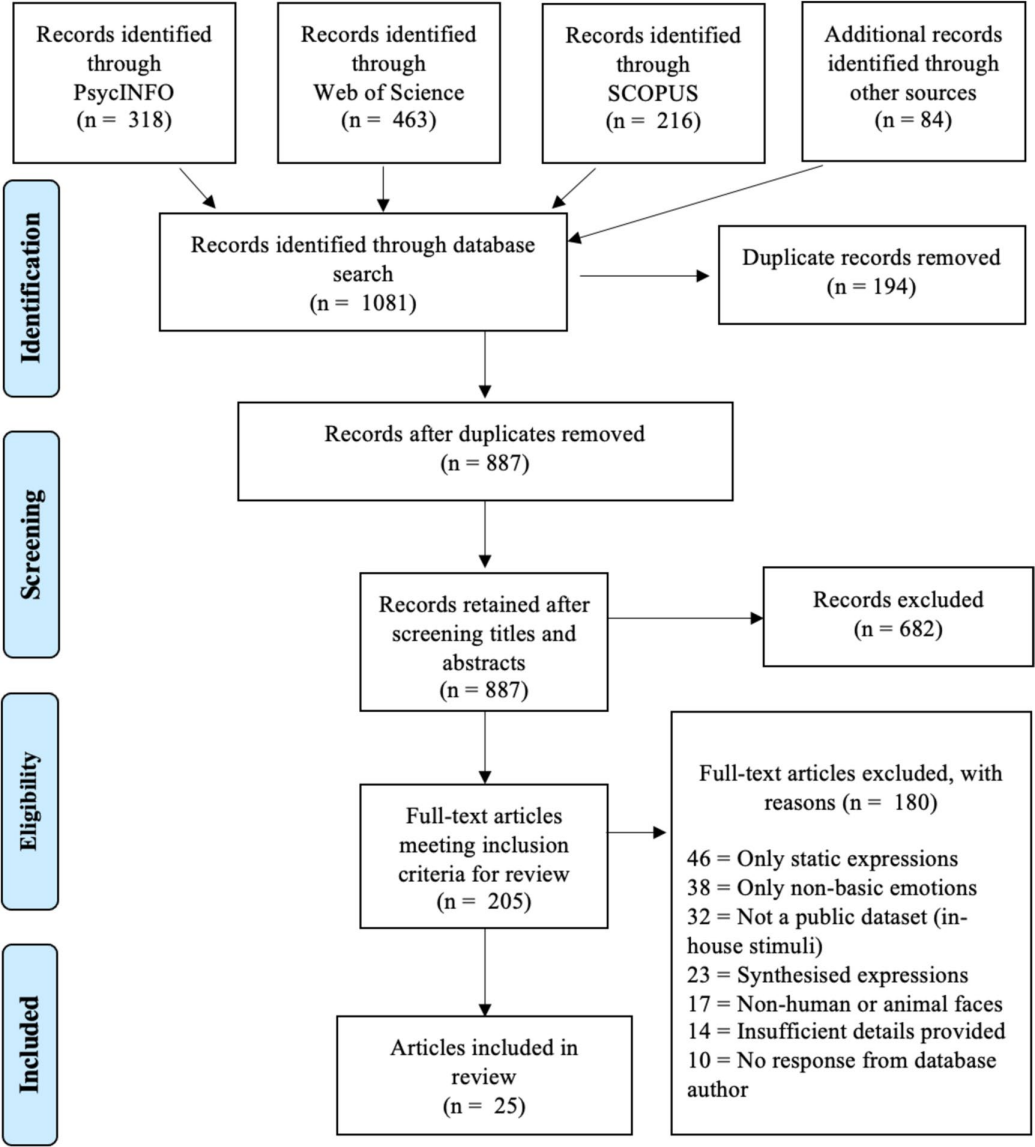
PRISMA flow diagram of literature search and selection procedure

The initial search yielded 1081 records, of which 318 were derived from *PsycINFO*, 463 from *Web of Science*, 216 from *SCOPUS* and 84 from other sources (Fig. 1). Duplicate entries were systematically removed, leaving 887 papers for title and abstract screening. Among those, 682 papers were deemed irrelevant, reducing the pool to 205 papers for a thorough full-text review. Following this in-depth analysis, we screened articles for eligibility. To be included in the systematic review, the following selection criteria were applied: (a) database article accessible and published between 1990 and 2023, (b) public accessibility of database, (c) spontaneous elicitation method (induction, autobiographical recall, mental simulation), (d) real human encoders, (e) facial stimuli with or without body gestures in visual or audio-visual modality, (f) a minimum of 4 basic emotions, (g) macro- (not micro-) expressions, and (h) dynamic stimuli in the form of videos or image sequences. One hundred eighty articles were disqualified for not meeting the predetermined inclusion criteria. The final list comprised 25 papers that qualified as publicly available spontaneous and dynamic FEDBs of basic emotions.

### Conceptual Features

This section reviews the thematic approaches that inform the development and validation of spontaneous and dynamic FEDBs. It aims to elucidate the conceptual features that characterise each database, i.e., emotional content and elicitation procedures, encoder demographics, and measurement and elicitation techniques. Table 2 summarises these key points, highlighting the scope and potential application of each database.

## Emotional Content and Elicitation

Among the reviewed stimulus sets, 15 databases primarily focus on basic emotions, often accompanied by a neutral face as a baseline expression. These databases classify emotions into discrete categories that are conceptually distinct (Ekman, 1994). The categorical approach ensures that each emotion stands out clearly (Calvo & Fernández-Martín, 2013), making this type of stimulus set particularly suitable for comparing recognition between different classes/families of emotions. In some databases, emotions such as anger and fear are omitted (e.g., DISFA, ISED, LIRIS-CSE) likely due to their difficulty of induction in the laboratory, and/or replaced by contempt (SAFE-FE).

Acknowledging the large variety of emotional states, 10 databases have broadened their scope beyond the six basic emotions (e.g., BP4D + , DynEmo, Emognition), integrating additional states like boredom and confusion to indicate various levels of engagement during expression elicitation. Some databases also include subtypes of emotions such as amusement, enthusiasm, and liking, thereby representing different degrees of arousal that might be overlooked if generic labels (i.e., happiness) alone were used (Russell, 1980). This approach enriches the emotional content of FEDBs, offering more diverse portrayals of emotions similar to those encountered in daily life (Calvo & D’Mello, 2010; Krumhuber et al., 2017).

With regard to the elicitation method, the majority of datasets utilise passive induction techniques using pre-selected emotional stimuli, such as images (e.g., International Affective Picture System; Lang et al., 2020) or videos (e.g., YouTube, TV shows, films). These stimuli, tailored to provoke an instant reaction from encoders, have garnered empirical support for their efficacy in emotion induction (Brave & Nass, 2009; Gross & Levenson, 1995; Schaefer et al., 2010), showing a robust alignment with encoders’ self-reported emotions, physiological responses, and neural correlates (for a review, see Siedlecka & Denson, 2019). Notably, whilst images offer a snapshot of affect-relevant moments, they may not encapsulate the dynamic progression of emotional events (Devilly & O’Donohue, 2021). In comparison, videos provide a multisensory experience through moving scenes, auditory cues, and emotional context, thereby conveying more immersive and holistic emotional narratives (Gross & Levenson, 1995). Consequently, they often evoke more intense and pronounced emotional reactions that are found to be stronger than those elicited by images (Horvat et al., 2015).

Both types of stimuli elicit facial expressions that closely resemble emotional responses in everyday life, whilst maintaining control over the recording environment (Coan & Allen, 2007). These databases frequently stand as the preferred choice of encoding, showcasing facial manifestations that resonate with authentic affective experiences (Zloteanu et al., 2018). This advantage further extends to decoding studies, where the recording protocol ensures consistent visibility of the face. Such uniformity serves as a benchmark for evaluating recognition accuracy in spontaneous expressions (Zhang et al., 2014).

Nonetheless, paradigms that rely solely on the viewing of visual stimuli can constrain the range of facial responses, overlooking the variability of real-world emotional experiences (Zupan & Eskritt, 2020). To address this potential limitation, several databases (e.g., BINED, BioVid, BP4D + , DynEmo) have diversified their elicitation techniques, incorporating interactive and actively engaging tasks (e.g., touching unknown objects in a box, smelling unpleasant odor, and playing games). These innovative approaches allow researchers to better capture complex emotional states such as secondary or self-conscious emotions (e.g., embarrassment, pride, and pain), resulting in a wider spectrum of emotions beyond the six basic emotions. Such expansion also facilitates a context-sensitive analysis of emotional reactions, where individuals may respond differently as a function of the situation, the environment, or the presence of other people (Hamann & Canli, 2004; Koval & Kuppens, 2012). These databases are particularly valuable for formulating and advancing deep-learning models of emotion recognition (for a review see Bian et al., 2023), which seek to enhance human–computer interaction through metadata about the emotion-inducing context (Sneddon et al., 2012).

## Encoder Demographics

Among the databases reviewed, the number of encoders varies considerably from a minimum of 7 (i.e., SPOS) to a maximum of 358 (i.e., DynEmo). Datasets with fewer encoders tend to incorporate more recordings per individual (e.g., SPOS, FEEDTUM), allowing for the exploration of intra-individual variability in emotion expressions. This approach is particularly valuable for examining the coherence between the experience and expression of emotion within an individual. In contrast, datasets with a larger subject pool (e.g., BINED, EB +) can better capture inter-individual variability in facial behavior. This is vital for developing affective computing systems that are robust to individual differences in facial features and generalise effectively to new faces. Despite the large variation in sample size, most databases show a preference for moderate numbers of encoders ranging from 40 to 60.

Another notable trend across datasets is the overrepresentation of young adults, possibly driven by the convenience of recruiting within academic settings. Merely a subset of databases contains a wider age span (e.g., BP4D-spontaneous, BP4D + , EB +). Whilst this trend might seemingly represent a demographic snapshot, it risks obscuring the influence of age on emotional expression including factors such as cognitive maturation/decline, muscle atrophy, and wrinkles (Houstis & Kiliaridis, 2009; Ko et al., 2021; Michaud et al., 2015). To compensate for this marked preference, certain databases have targeted specific age groups like children (ChildEFES, LIRIS-CSE); nonetheless, the underrepresentation of the elderly population persists.

Most databases have a fairly equal representation of male and female encoders, although some are slightly skewed towards one gender group (e.g., NVIE, UT-Dallas). Balanced gender ratios are paramount for stimulus development to reflect known differences in emotion processing between the sexes (Wiswesser et al., 2018). Despite efforts to incorporate diverse ethnic backgrounds, there remains a skewed focus on White/Caucasian and Asian encoders, likely reflecting the geographical location of data acquisition. Whilst this approach provides valuable insights into culture-specific differences in expression, it poses challenges to the broader cross-cultural generalisability of a dataset. The representation of diverse ethnic backgrounds is of particular importance for spontaneous facial expressions, which may be subject to cultural dialects (Cordaro et al., 2018; Elfenbein et al., 2007).

## Emotion Measurement and Validation

Data annotation refers to the process of adding descriptive information or labels to stimuli for specifying the emotional content of recordings. Annotation is a labor- and time-intensive task that demands considerable effort by the experimenter. This process might be further complicated by the subtlety and complexity of spontaneous expressions. Having well-annotated videos of facial behavior considerably amplifies the value of a database, especially for affective computing research which relies on the training and testing of machine algorithms (Zhang et al., 2014).

Most databases provide annotation to some extent, serving as empirical ground truth for facial expressions. These commonly adhere to predefined emotion categories (i.e., six basic emotions) and align with the emotion-provoking stimulus designed to elicit an affective reaction (Lucey et al., 2010). Whilst access to well-labelled data enables a systematic and validated framework for interpretation, annotation in terms of only one emotion category can be problematic, especially when there is an inconsistency between what is shown on the screen (e.g., an amusing scene) and what is experienced by the encoder (e.g., surprise, disgust). To avoid the potential risks of oversimplifying multifaceted emotional states, it is imperative to treat data labels with caution (Fanelli et al., 2010).

Here, the Facial Action Coding System (FACS; Ekman et al., 2002) might prove useful for analysing subtle and complex facial configurations. Eight databases have adopted FACS coding to comprehensively quantify facial behavior in terms of action units (AUs). Rather than relying on a limited set of predefined emotion labels, FACS directly measures observable AUs without presumptions about the underlying affective state. This enables greater flexibility in interpreting facial behavior from a broader perspective. Also, researchers can refer to the extensive empirical evidence linking AUs to a wide range of affective states that might otherwise be overlooked by fixed emotion categories.

To validate the emotional content of recordings, most FEDBs provide self-reports of subjective states as a resource for stimulus assessment. Introspective techniques allow encoders to articulate their feelings, offering a convenient, albeit not always reliable (Cowen & Keltner, 2017), window into a person’s emotional experiences. These methods frequently employ categorical ratings, enabling individuals to classify their feelings into distinct sets such as the six basic emotions. Some databases (e.g., BioVid, Emognition, PPB-Emo) have integrated continuous ratings in which affective states are mapped onto dimensional spaces including valence and arousal (and dominance) to capture subtle variations and gradations inherent in affective states (Russell, 1980). Some databases (e.g., PEDFE, DynEmo) further measure emotion genuineness and action readiness (e.g. approach, avoidance), offering valuable insights for evaluating expression authenticity and behavioral intentions.

The emotional content of recordings can also be validated by external inferences from human observers or machine recognition. Consensus judgments by naïve observers, who are asked to classify expressions, are typically considered to be more objective because they are less prone to social desirability and memory biases. Their inclusion also emphasises the human ability to recognise subtle nuances in emotional content common to spontaneous facial behavior (Krumhuber et al., 2021; Yitzhak et al., 2017). Nonetheless, the cost and effort associated with collecting data from human participants can be large. For this reason, AFEA stands out as the primary evaluation method across most databases (e.g., EB + , Emognition, iSAFE), offering a standardised and efficient approach for processing large amounts of data. AFEA can provide both discrete and continuous ratings of emotions and AUs. Despite the advantages of AFEA, human annotation remains valuable for validating algorithms and addressing challenges that automated approaches cannot yet handle well (e.g., detection of subtle facial movements, co-occurrence of multiple AUs).

For all validation techniques, it is crucial to note that an over-reliance on any single approach for measuring or labelling facial behavior can be problematic. Emotions represent multifaceted processes that cannot be adequately processed by a single system (Gross & John, 1997). For instance, inconsistencies may emerge in how emotions are experienced, expressed, or perceived due to social norms that down- or up-regulate different components of emotional responses (Bonanno & Keltner, 2004; Elfenbein & Eisenkraft, 2010). To avoid oversimplifying the intricate nature of emotional experiences, it is imperative to utilise and integrate multiple, complementary methods to thoroughly measure and validate the relations between emotions and facial behavior. Some databases (e.g., PEDFE, iSAFE) have adopted such a comprehensive framework by incorporating self-report data, observer ratings, and FACS coding, which maximise the potential utility of FEDBs.

### Technical Features

This section reviews the technical features of FEDBs, i.e., stimulus numbers and duration, frame rate, and resolution. These features are crucial for the utilisation of FEDBs in human studies as well as for human–computer interaction. Table 3 provides a summary of these technical features for each dataset.

Databases vary considerably in the number of captured expressions, with the total amount of recordings ranging from as few as 147 (i.e., SPOS) to as many as 1400 (i.e., BINED). Overall, most databases depict high numbers of recordings (approximately 400 to 700), indicating a wide spectrum of facial expressions being captured. Large stimulus numbers are particularly important for the training and testing of computer models sufficiently robust to stimulus variations. They can also act as a benchmark for comparing different expression recognition algorithms (Turk & Pentland, 1991; Valstar et al., 2015, 2017).

With regard to the recording quality, many databases utilise medium (720 × 480 pixels) to high-resolution (1920 × 1080 pixels) cameras. The latter captures more detailed changes in the morphological features (e.g., shapes and textures) of facial behavior, which is crucial for the accurate representation of emotional states (Li et al., 2022). Nominal resolution is particularly important for fine-grained analysis of spontaneous expressions, which often manifest heterogeneous and subtle facial configurations (Namba et al., 2017; Pfister et al., 2011).

Frame rate refers to the number of frames recorded per second (fps). The higher the frame rate, the smoother and more fluid the facial movement appears to be. Whilst many databases use frame rates of 25 and 30 fps, some databases (e.g. Emognition, SASE-FE) adopt notably higher frame rates (60 to 100 fps). A higher frame rate is preferred for capturing and analysing the dynamic trajectory of spontaneous displays (Krumhuber et al., 2023), which provides a more vivid depiction of how facial expressions unfold and progress over time (Leonard et al., 1991). The enhanced temporal resolution also facilitates the identification of rapid and transient facial movements that might be easily missed at lower frame rates.

Lastly, there is variability across databases in the duration of portrayals, ranging from 0.5 s (i.e., BAUM-1) to 4 min (i.e., BioVid, DISFA). Extended durations intuitively offer more information. However, long videos can also encompass periods devoid of emotional content, especially if the encoder is recorded throughout the entire elicitation task. Such non-emotive periods can introduce noise to the data, potentially complicating the analysis and recognition of facial expressions. For this reason, most databases have segmented their recordings, thereby concentrating on key expressive phases from onset to apex and offset.

### Practical Features

Committing to open science principles promotes knowledge sharing and offers long-term benefits for future research. To facilitate the utility of publicly available stimulus sets, this section provides practical information about dataset accessibility whilst emphasising ethical compliance for data usage. Table 4 summarises information on how to access the datasets and potential ethical restrictions to be considered.

Most databases provide access through a dedicated website link. These platforms often detail the database’s key features and offer additional information (e.g., experimental manipulation, and annotation) beyond the published article. In addition, the author’s email address serves as an initial point of contact for inquiries about the database. We contacted all database authors via email to confirm public availability of their stimulus sets.

Researchers also adopted various practices to ensure the ongoing distribution of datasets whilst safeguarding the responsible and ethical usage of datasets. Many databases mandate a signed End User License Agreement (EULA) to protect participants’ rights and prevent potential data misuse. As such, most datasets are restricted to academic research purposes only, with additional consents required for commercial use (e.g., BP4D). These EULA forms can be accessed through the website links or by directly contacting database authors through the email address listed in Table 4.

Moreover, some databases are directly distributed through the Open Science Framework (OSF) platform without requiring any EULA. This practice streamlines data acquisition by removing administrative barriers and bypassing lengthy processes of obtaining ethical approval. However, some databases (e.g., BP4D-Spontaneous, BP4D + , EB +) impose handling fees for data maintenance and delivery for over 10 TB data.

### General Discussion

In the last two decades, there has been a major shift in basic emotion research towards more ecologically valid facial stimuli (Krumhuber et al., 2023). Unlike posed displays depicting highly standardised/prototypical portrayals to maximise their recognisability, spontaneous expressions do not involve fixed signals of emotion (Parkinson, 2013), making them more variable but representative of affective responses seen in real life. Such growing interest in stimulus validity has notably accelerated the development of FEDBs, with a number of papers surveying existing corpora (e.g., Diconne et al., 2022; Guerdelli et al., 2022; Haamer et al., 2018; Siddiqui, 2022; Weber et al., 2018). Yet, a systematic and topical review focusing on basic emotions portrayed exclusively by spontaneous, dynamic expressions is currently missing. The present paper aims to fill that gap with the ultimate purpose to assist readers in their decisions about stimulus selection.

Among all FEDBs, there is a clear trend towards a broader range of emotion categories beyond the basic six, thereby acknowledging the complexity and diversity of human experiences. Such expansion not only enriches the theoretical understanding of emotions but also holds practical significance for AFEA in terms of its ability to generalise to a wider spectrum of everyday emotional phenomena (Bänzinger et al., 2011; Gunes & Pantic, 2010). Although some FEDBs incorporate a mixture of elicitation techniques (active, passive, interactive), the majority rely on (audio-) visual materials (i.e., images, films, video-clips) for emotion induction. Unlike naturalistic ‘in-the-wild’ displays recorded in unstructured/unconstrained environments, spontaneous expressions are evoked under experimental conditions. The stimulus presentation ensures consistent and replicable responses across senders. However, it also limits the number of emotion-inducing situations typically experienced in real life. Future work may aim for greater variety in experimental methods for inducing basic emotions, also piloting materials/tasks for their effectiveness in evoking the relevant emotional state.

Many FEDBs feature a moderate number of encoders, with a relatively equal gender balance, although a notable focus on young adults as well as White/Caucasian and Asian encoders persists. For the training and testing of computer models, it will be important to collect large amounts of data from diverse demographics. Also, stimulus sets with higher temporal resolution (> 30 fps) are needed for capturing rapid facial movements.

Besides sender-relevant characteristics, it is important to note that not all basic emotions are equally easy to elicit using experimental methods. For example, anger may be difficult to induce in a controlled setting (Siedlecka & Denson, 2019), particularly when participants are aware that they are being filmed. In addition, facial behavior obtained in the laboratory may be restricted in motion due to fixed camera positions. In the future, recording conditions could be less constrained by filming in natural environments (with multiple and hidden cameras) that allow for greater privacy, without compromising the experimental control in data acquisition (i.e., noise level, illumination, and occlusion).

Whilst induction materials are effective in eliciting the target emotion, it is possible that more than one emotion is felt by the encoder. Moreover, there may be considerable variability in how individuals appraise and respond to the stimulus content. At the moment, database validation approaches rely mainly on categorical emotion labels, which fail to capture subtle differences in cognitive and affective dimensions of emotion. Future research is needed to provide more fine-grained labels, thereby utilising both categorical and dimensional approaches to capture variability in emotional experiences (Cowen et al., 2021). This may also include meta-data such as audio and physiological signals (e.g., heart rate, skin conductance) for gaining complementary insights into emotional states (Jerritta et al., 2011; Juslin & Laukka, 2003), particularly when those are mixed.

Among all validation efforts, AFEA stands out as the predominant method. The trend likely stems from the overarching goal of many FEDBs to refine existing computer algorithms, with the potential to improve human–machine interaction. This focus has solidified AFEA as a reliable tool for facial expression recognition (Lewinski et al., 2014), often comparable to or even surpassing human performance (Del Líbano et al., 2018; Krumhuber et al., 2021; Lewinski et al., 2014). It should be noted though that their accuracy hinges on the integrity of the training data and the robustness of the underlying algorithms (Koelstra et al., 2010). Specifically, classifiers often train on specific segments of a database, reserving the rest for testing (Gupta et al., 2016). Also, most past efforts hinge on proprietary in-house algorithmic models which may not be easily accessible for cross-laboratory research (Dupré et al., 2020). In the future, it will be important to conduct cross-classifier and cross-corpus validations to allow for greater transparency in database assessment. The present article provides a first step in comparatively evaluating multiple spontaneous and dynamic FEDBs of basic emotions, thereby highlighting their commonalities and differences.

## Additional Information

### Data Availability

No datasets were generated or analysed during the current study.
